# Composite Membranes of Poly(ε-caprolactone) with Bisphosphonate-Loaded Bioactive Glasses for Potential Bone Tissue Engineering Applications

**DOI:** 10.3390/molecules24173067

**Published:** 2019-08-23

**Authors:** Zoi Terzopoulou, Diana Baciu, Eleni Gounari, Theodore Steriotis, Georgia Charalambopoulou, Dimitrios Tzetzis, Dimitrios Bikiaris

**Affiliations:** 1 Laboratory of Polymer Chemistry and Technology, Department of Chemistry, Aristotle University of Thessaloniki, GR54124 Thessaloniki, Central Macedonia, Greece; 2 National Center for Scientific Research “Demokritos”, GR15341 Athens, Ag. Paraskevi Attikis, Greece; 3 Biohellenika Biotechnology Company, Leoforos Georgikis Scholis 65, GR57001 Thessaloniki, Central Macedonia, Greece; 4 School of Science and Technology, International Hellenic University, GR57001 Thermi, Central Macedonia, Greece

**Keywords:** bioactive glasses, poly (ε-caprolactone), nanocomposites, strontium, ion release, biocompatibility, osteogenesis, bone tissue engineering

## Abstract

Poly(ε-caprolactone) (PCL) is a bioresorbable synthetic polyester with numerous biomedical applications. PCL membranes show great potential in guided tissue regeneration because they are biocompatible, occlusive and space maintaining, but lack osteoconductivity. Therefore, two different types of mesoporous bioactive glasses (SiO_2_-CaO-P_2_O_5_ and SiO_2_-SrO-P_2_O_5_) were synthesized and incorporated in PCL thin membranes by spin coating. To enhance the osteogenic effect of resulting membranes, the bioglasses were loaded with the bisphosphonate drug ibandronate prior to their incorporation in the polymeric matrix. The effect of the composition of the bioglasses as well as the presence of absorbed ibandronate on the physicochemical, cell attachment and differentiation properties of the PCL membranes was evaluated. Both fillers led to a decrease of the crystallinity of PCL, along with an increase in its hydrophilicity and a noticeable increase in its bioactivity. Bioactivity was further increased in the presence of a Sr substituted bioglass loaded with ibandronate. The membranes exhibited excellent biocompatibility upon estimation of their cytotoxicity on Wharton’s Jelly Mesenchymal Stromal Cells (WJ-SCs), while they presented higher osteogenic potential in comparison with neat PCL after WJ-SCs induced differentiation towards bone cells, which was enhanced by a possible synergistic effect of Sr and ibandronate.

## 1. Introduction

Guided tissue/bone regeneration (GTR/GBR) with barrier membranes is a commonly used approach for enhanced bone regeneration, especially during periodontal defect healing [1]. These membranes act as an occlusive barrier that blocks cell migration from connective tissue and epithelium to the defect, which can interfere with tissue regeneration and consequently provides the tissue with adequate time to heal [2,3]. Ideally, a GTR/GBR membrane is biocompatible, non-immunogenic, non-toxic, biodegradable after mechanically supporting tissue formation and able to stimulate cellular exclusion at the affected location and maintain a space for the surrounding healthy tissue to migrate [4,5].

Commercially available GTR/GBR membranes are either non-resorbable (polytetrafluoro-ethylene), or resorbable (polylactide (PLA), polylactide-co-glycolide (PLGA), polylactide-co-caprolactone, collagen) [5]. While non-resorbable membranes show excellent biocompatibility, they do require an additional surgical procedure for their removal and may cause soft tissue dehiscence [6]. These complications resulted in the commercialization of resorbable membranes that are however still constructed of bioinert polymers that lack osteoconductive/osteoinductive properties. Poly(ɛ-caprolactone) (PCL) has attracted a lot of attention due to its excellent biocompatibility, good biodegradability, easy handling, sterilization capability, inexpensive manufacturing process and control over the biodegradation rate, which resulted in its FDA approval for several clinical uses [7]. Compared with the most commonly used PLA and PLGA, PCL doesn’t release acidic degradation products that could affect cell growth [4]. Although PCL has many advantages, only a handful of studies have been published concerning PCL based membranes for GTR/GBR [8,9,10,11,12,13,14,15], as it shows low bioactivity, high hydrophobicity and weak mechanical properties that limit its effectiveness [15,16].

A commonly used method to increase bioactivity, alter the degradation rate, improve mechanical properties and enhance cell adhesion and proliferation on polymeric surfaces intended for tissue engineering applications including GTR/GBR [11,15,17,18,19,20,21,22,23,24,25,26,27,28,29,30], is the incorporation of bioactive inorganic fillers, like hydroxyapatite, tricalcium phosphate or bioglasses (BGs) [31,32,33,34,35,36,37]. BGs consist of a silicon dioxide-based network modified by other metal oxides such as Na_2_O, CaO and P_2_O_5_ [38]. They are among the most used synthetic materials in bone tissue engineering applications due to their excellent biocompatibility as well as their ability to stimulate osteogenesis and angiogenesis [39,40]. Several inorganic ions are shown to stimulate bone formation, with Ca and P being essential for bone mineralization as they comprise the main elements of hydroxyapatite [HA, Ca_10_(PO_4_)_6_(OH)_2_] [41]. Recently, the impact of other ions including Si, Zn, Mg, Sr, Ti, B and Cu on bone formation has been reported [42], that led to an increasing number of studies on BGs with different substitutions [43]. Among them, Sr shows beneficial effects on bone cells and for bone formation in vivo and for osteogenic differentiation at low doses [44,45,46,47,48,49,50,51]. Another approach to provide occlusive GTR membranes with the ability to regenerate bone is the incorporation of growth factors, drugs or other biomolecules [5,52,53]. However, the physical adsorption of biomolecules on polymeric membranes has the drawbacks of low immobilization efficiency and fast desorption which make their covalent bonding on the polymer necessary [53]. To overcome these problems, BGs can be used as both fillers and drug carriers in order to provide the membranes with bioactivity and hydrophilicity and additionally act as vehicles for the encapsulation of a drug molecule [18,54,55]. The adsorption of the drug or other biomolecules which can usually be quite unstable can both protect these molecules and control their release rate. Furthermore, since osteoconductive drugs and biomolecules are predominantly hydrophilic, their addition in hydrophobic polymeric films like PCL would be unsuccessful.

The combination of bisphosphonate (BP) drugs like alendronate and clodronate with bioglass, results in hybrid particles with a stronger capacity to regenerate bone defects than bioglass on its own [56,57]. It was also hypothesized that Sr substituted bioglass would further boost the osteogenic potential of BP-BG hybrid particles [56]. Potent osteoinductive drugs like BPs are used for treating various skeletal disorders, like osteoporosis, tumor-associated osteolysis, hypercalcemia and Paget’s disease [58,59,60]. BPs enhance osteoblast proliferation and maturation, inhibit osteoblast apoptosis and increase bone formation [61]. The clinical pharmacology of BPs revealed that their affinity to hydroxyapatite is the basis for their use as inhibitors of ectopic calcification and bone resorption [59]. Since their bioavailability when administered orally is very low (1–6%), localized delivery has been studied and it was reported that local delivery of BPs can lead to an improvement in bone growth around implants [62].

In our previous work, we demonstrated that the incorporation of 0.5, 1 and 2.5 wt% of nanosized mesoporous BGs in a PCL matrix via the in-situ ring opening polymerization of ε-caprolactone, resulted in a biocompatible PCL composite material with superior mechanical properties, accelerated biodegradation rate and bioactivity [63]. In this context, PCL/BG composites and nanocomposites are promising materials for a wide range of applications such as coatings for metallic or ceramic scaffolds for bone regeneration [64,65,66], as a solution for their high corrosion rates or low bioactivity, as root canal filling materials, or as bone scaffolds [15,63,67,68,69,70].

The aim of the present work was the development of BP-loaded composite thin PCL membranes with two mesoporous BGs of different compositions with improved characteristics for potential use as GTR/GBR membranes. The novel combination of the exceptional bioactivity of BGs with the capacity of BPs to strongly coordinate Ca^2+^ ions is expected to transform PCL to a highly bioactive and biocompatible membrane, suitable for GBR. Two types of mesoporous ternary BGs (SiO_2_-CaO-P_2_O_5_ and SiO_2_-SrO-P_2_O_5_) were synthesized with a hydrothermal method aiming to investigate the effects of fully replacing Ca with Sr. Afterwards, the BP drug ibandronate (Iba) was loaded on the BGs. These BG fillers were finally incorporated at a concentration of 10 wt% in PCL films by spin coating. The obtained thin films were studied in terms of morphology, dispersion, thermal properties, mechanical properties, ion release, hydrophilicity, cell viability and the combined effect of the ions Ca, P, Sr and Iba on the bioactivity and osteogenic differentiation potential of the PCL membranes was evaluated.

## 2. Results and Discussion

### 2.1. Characterization of the Bioglasses

SrO/CaO substitution has emerged as a new method in designing new bioactive materials for bone repair and regeneration [45]. Sr and Ca are similar in chemical nature in terms of ionic radius and charge, and the substitution of Ca with Sr does not considerably alter the basic chemical and physical properties of Ca-containing BGs and ceramics such as Bioglass^®^, hydroxyapatite, and calcium phosphates. Sr however has a lower charge to size ratio that results in the formation of a looser glass network [44].

**Figure 1:**
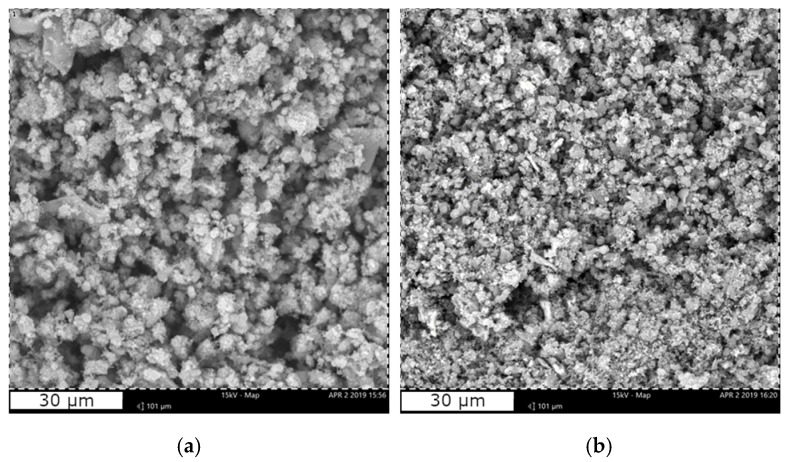
SEM micrographs of (**a**) CaBG and (**b**) SrBG bioglass particles.

SEM images of the as-produced CaBG and SrBG BGs (Figure 1) show that the hydrothermal method employed and the calcination at 600 °C leads to the formation of aggregates of nanoparticles in both cases. DLS results (Table S1) report sizes of 235.7 ± 26.11 nm for CaBG and 408.3 ± 71.28 nm for SrBG. Their wide particle size could be attributed to the agglomeration of smaller nanoparticles during the crystal growth process [71]. According to the literature [72], increase of the CaO content in mesoporous bioglass nanoparticles results in decreased particle size. In contrast, increase of Sr^2+^ content was found to lead to a significant increase of the average particle size due to aggregation and coalescence of clusters [73]. The EDX elemental analysis (Table 1) confirmed the presence of O, Si, P and Ca in CaBG and O, Si, P and Sr in SrBG.

**Figure 2:**
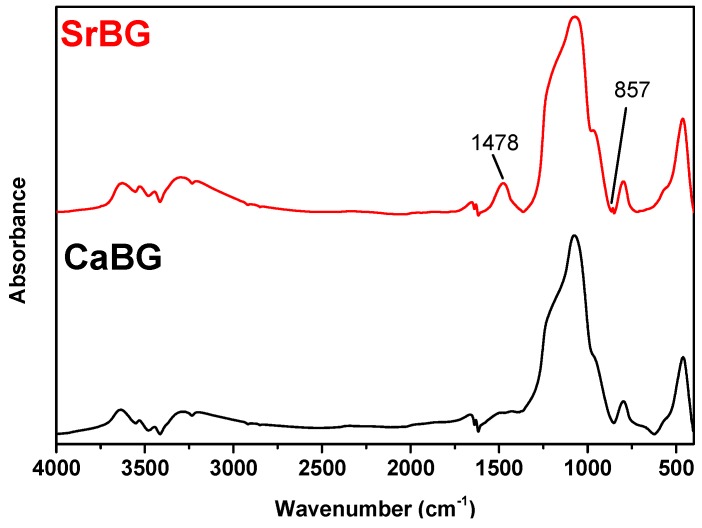
FTIR spectra of CaBG and SrBG BGs.

FTIR spectra of both BGs are presented in Figure 2. Both samples exhibit mainly the stretching and bending vibrations of the Si–O–Si bridge. Characteristic bands between 400 and 500 cm^−1^ are assigned to the bending vibrations of the Si–O–Si and O–Si–O bonds [74,75]. The peak in the range of 760–810 cm^−1^ corresponds to the stretching vibrations of the O–Si–O bonds, while the peak in the range of 1000–1100 cm^−1^ is attributed to the symmetric stretching vibration of the Si–O–Si bonds [74]. The absorption peak at 1635 cm^−1^ is associated with the stretching mode of the OH group [75]. In addition, a weak band at cc. 570 cm^−1^ can be attributed to the asymmetric vibration of PO_4_^3−^ [76]. The FTIR spectrum of SrBG exhibits two additional peaks around 1478 cm^−1^ and 857 cm^−1^ that can be attributed to C–O stretching in carbonate groups (CO_3_^2−^), which can be formed by the reaction of SrBG with atmospheric CO_2_ [75,77,78]. No peaks that can be assigned to organic matter are observed, confirming the purity of the materials.

**Figure 3:**
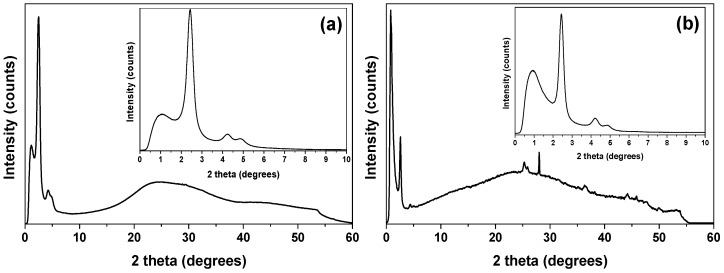
Wide- and low-angle XRD patterns of (**a**) CaBG and (**b**) SrBG BGs.

The wide-angle XRD pattern of the calcined CaBG material depicted in Figure 3a confirms its amorphous nature, in agreement with previous studies [79]. The pattern of the calcined SrBG, depicted in Figure 3b, shows some small peaks which are related to strontium silicate (Sr_2_SiO_4_) and strontium carbonate (SrCO_3_). The presence of strontium crystalline compounds like these in bioactive glasses was also reported by Solgi et al., who found out that the amorphous structure of bioactive glasses is altered by the incorporation of strontium [80,81]. In the low angle region (Figure 3, insets) the XRD patterns of both CaBG and SrBG show three diffraction peaks at 2θ ca. 2.4, 4.2 and 4.9° which can be well indexed as (100), (110), and (200) reflections of the P6 mm hexagonal symmetry, and this suggests a well-ordered arrangement of pores in the prepared BGs [82,83,84].

**Figure 4:**
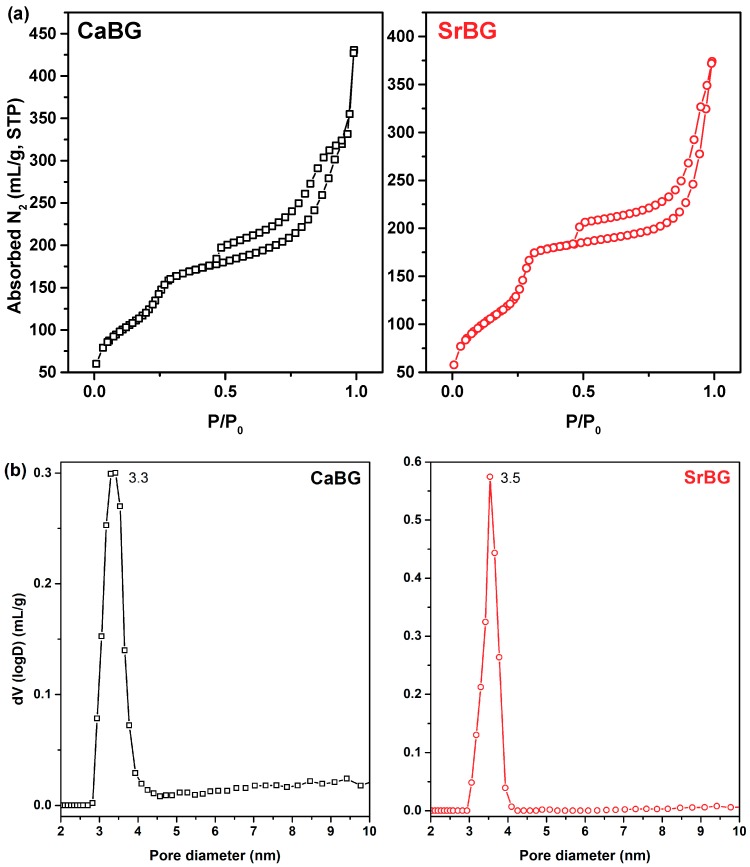
(**a**) N_2_ adsorption-desorption isotherms and (**b**) respective pore size distributions of CaBG and SrBG BGs.

The pore properties of the calcined CaBG and SrBG samples were determined by analysis of their corresponding N_2_ adsorption/desorption isotherms at 77 K (Figure 4a,b), which, according to the classification of IUPAC are type IV, characteristic of mesoporous materials.

Overall CaBG was found to have a BET area of 588 m^2^/g and a total pore volume of 0.51 cm^3^/g (the latter calculated at P/P_0_ = 0.98); the respective values for SrBG were 552 m^2^/g and 0.5 cm^3^/g (at P/P_0_ = 0.98). In addition, the materials showed a mean pore size around 3.3–3.5 nm for the CaBG and SrBG, respectively, according to the deduced pore size distributions (Figure 4c,d). The obtained specific surface area values are comparable to previous studies [85,86] and remarkably higher than those of conventional sol-gel glasses, allowing an improved reactivity in physiologic environment [87,88].

**Figure 5:**
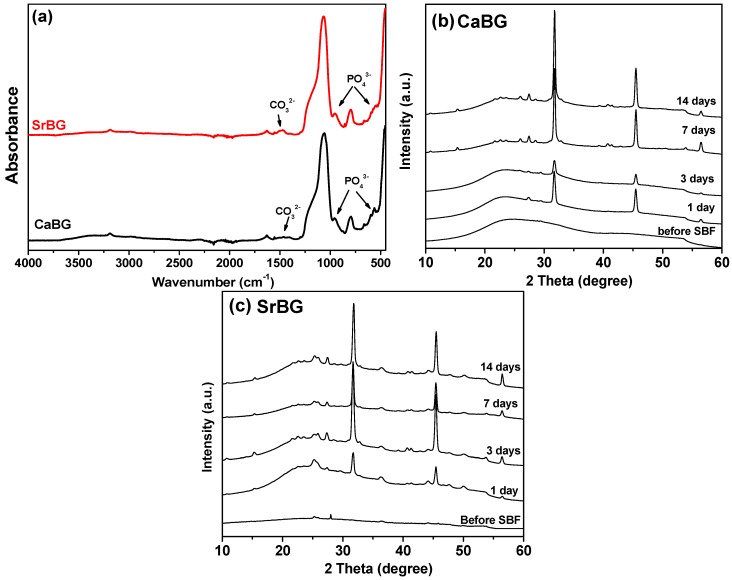
(**a**) FTIR spectra of the CaBG and SrBG BGs after immersion in SBF for 14 days, XRD patterns of (**b**) CaBG and (**c**) SrBG after immersion in SBF solution for 14 days.

Bioactivity of the BGs was assessed after immersion in SBF for 14 days. The FTIR spectra obtained are presented in Figure 5a. New absorption bands were observed around 540–601 cm^−1^, 958 cm^−1^ and 1058 cm^−1^, which are characteristic of hydroxyapatite [75,89]. More specifically, the peaks at 540–601 cm^−1^ and 958 cm^−1^ are associated with the P–O bending mode of crystalline phosphate (apatite) deposited on the glasses, while the absorption band at around 1058 cm^−1^ is associated with the P–O stretching mode [90]. Additionally, two peaks around 870 and 1401–1473 cm^−1^ attributed to C–O stretching in carbonate groups were also observed [75]. The intensity of the peak around 1635 cm^−1^ increased after 14 days, which was attributed to the OH stretching mode [75]. The presence of CO_3_^2−^, PO_4_^3−^ and OH signals in the FTIR pattern can confirm the formation of hydroxyapatite on the surface of glasses after 14 days.

The XRD patterns of the CaBG and SrBG BGs after immersion in SBF solution for 1, 3, 7, and 14 days (Figure 5a,b) show characteristic peaks of hydroxyapatite and strontium apatite, verifying their bioactivity. Specifically, after soaking for one day, the patterns of both glasses show the existence of apatite peaks at 2θ = 25.9°, 2θ = 27.3°, 2θ = 31.8°.

Nevertheless, it is observed that SrBG exhibits more and better visible features, especially in the area of 2θ = 25.9°, 2θ = 27.3° (Figure 5c) compared to CaBG. These results indicate the accelerating effect of Sr ions on the apatite formation ability of BGs, in agreement with previous studies on other BG systems [75,78,80,81,89,90].

**Figure 6:**
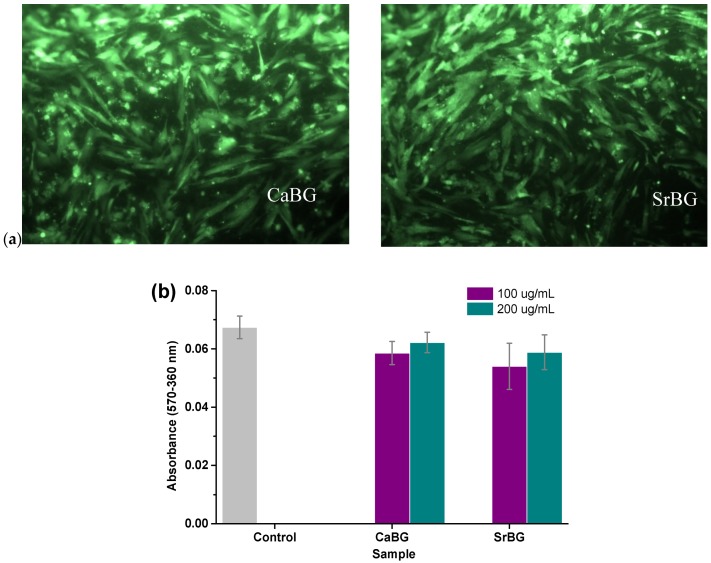
(**a**) Fluorescence microscopy micrographs of WJ-MSCs cells after exposure to CaBG and SrBG particles in the medium and (**b**) MTT assay results of WJ-MSCs cells after seeding for 24 h on CaBG and SrBG.

The in vitro biocompatibility of CaBG and SrBG in two different concentrations in the cell culture medium (100 ug/mL and 200 ug/mL) was tested by MTT assay, after seeding WJ-SCs on tissue culture polystyrene. After 24 h, the viability of the cells was evaluated with fluorescence microscopy images (Figure 6a). The bright green spots are the viable cells that prove their successful adhesion and proliferation on both bioglasses. MTT assay results (Figure 6b) support the findings of the fluorescence microscopy results. More specifically, neither of the BGs affect the metabolic activity significantly, since the absorbance values are very close to that of the control WJ-SCs (*p* > 0.05). In the fluorescence microscopy micrographs, it appears that the cells proliferated continuously on the surface of the samples and formed cell-cell interactions and eventually a homogeneous cell layer.

#### Adsorption of Iba on the Bioglasses

Mesoporous bioactive glasses with well-ordered mesopore structures, large surface area and pore sizes ranging from 5 to 20 nm that can be synthesized by sol-gel and hydrothermal methods can be easily loaded with different active compounds, making them ideal candidates for controlled drug delivery [91].

A preliminary in vivo study reported that the combination of the BP alendronate with BG led to increased bone formation on rats in comparison with the control groups [92]. A few years later, Rosenqvist et al. explored the physicochemical interactions of clodronate with BG based on the hypothesis that their beneficial impact originates not only from the pharmacological properties of the drug but also from the formation of a complex between them that creates a favorable environment for ion exchange [57].

**Figure 7:**
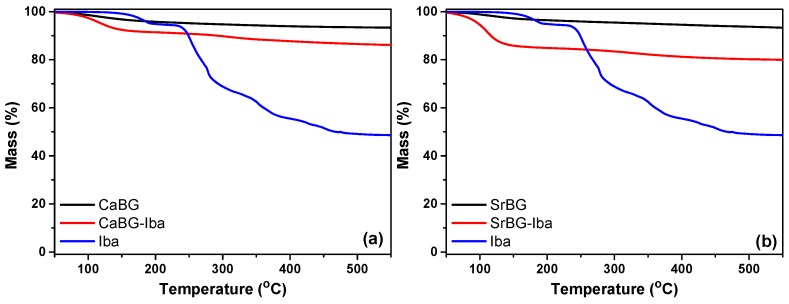
TGA thermograms of Iba and (**a**) CaBG, (**b**) SrBG before and after Iba adsorption.

The amount of Iba adsorbed on the pristine BGs was determined by TGA. The thermal stability of the as produced CaBG and SrBG BGs after calcination was confirmed by TGA and the results are presented in Figure 7. Both BGs exhibit two main weight loss steps. The first one with a T_max_ at 105 °C corresponds to loss of about 4.2 and 3.5% physically adsorbed water for CaBG and SrBG, respectively. The second step occurs in the temperature range 200–800 °C. In general, both glasses are very thermally stable as expected, and lose 8% of their initial mass when heated to 800 °C.

The thermal degradation of Iba occurs in multiple stages (180 °C, 254 °C, 276 °C, 353 °C, 423 °C, 452 °C), while at 795 °C there is a solid residue of 47.8%. Based on the difference in mass loss of the BGs with and without Iba, the amount of adsorbed drug was calculated at 3.78 wt% and 8.41 wt% for CaBG and SrBG, respectively. Similar drug loading adsorption capacities were reported in the bibliography for alendronate [93]. The drug loading capacity of SrBG is larger probably due to its slightly larger pores and the expansion of the glass network caused by the larger Sr ions compared to Ca.

**Figure 8:**
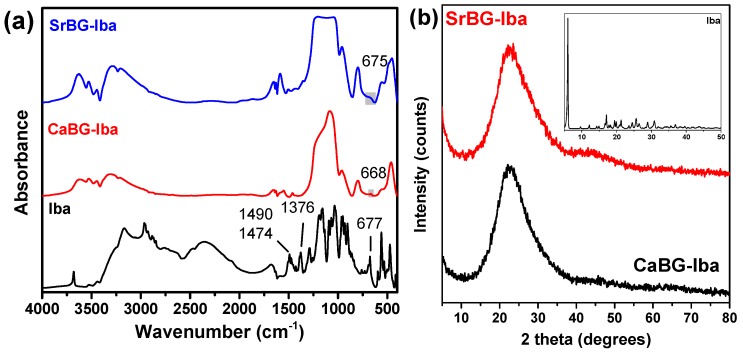
(**a**) FT-IR spectra of CaBG-Iba and SrBG-Iba and (**b**) X-ray diffraction patterns of Iba, CaBG-Iba and SrBG-Iba.

Figure 8a shows the FT-IR spectra of Iba and CaBG and SrBG after Iba adsorption. The spectrum of Iba has a multitude of peaks; those in the spectral range 1200–900 cm^−1^ are due to the bending vibrations of the CO and P=O groups [94], the peak at 1376 cm^−1^ is due to the vibration deformation of the methyl groups and the peak at 667 cm^−1^ is probably due to some type of stretching vibration of the phosphate groups [95].

After the incorporation of the drug, the FTIR spectra of the BGs present with some differences. The 677 cm^−1^ peak of Iba appears slightly shifted in the spectra of CaBG-Iba and SrBG-Iba, to 668 cm^−1^ and 675 cm^−1^ respectively, indicating the adsorption of the drug through the formation of strong bonds between the BGs and the phosphate groups of Iba.

XRD was used in order to examine how crystallinity of Iba is affected by its incorporation in the BGs. Iba itself shows many crystalline reflections, with the main located at 2θ = 6°, while it is known that it is a polymorphic form [96]. However, the patterns of the CaBG-Iba and SrBG-Iba show only one broad halo, as can be seen in Figure 8b, indicating that the adsorbed drug is in amorphous state. DSC thermograms of CaBG-Iba and SrBG-Iba during heating are presented in Figure S1 where no peak of Iba was detected, suggesting its amorphization, in agreement with the XRD results.

### 2.2. Characterization of Composite PCL Thin Films

#### 2.2.1. Morphological Characterization

The basic methods used for the preparation of GTR membranes are solution casting, spin coating and electrospinning [5]. Spin coating is an inexpensive technique for uniform thin film fabrication that allows easy control of film thickness by altering the polymer concentration in the solution used [97]. All the prepared membranes covered the entire surface of the glass substrates homogenously and had a hazy appearance, suggesting that crystallization occurred during the spinning process. The thickness of the composite PCL/bioglass (with and without Iba) films deposited by spin coating on the glass slides was approximately 7.5–8 μm (whereas the neat PCL films were around 9 μm). The average mass and thickness values of the prepared membranes can be found in Table S2.

**Figure 9:**
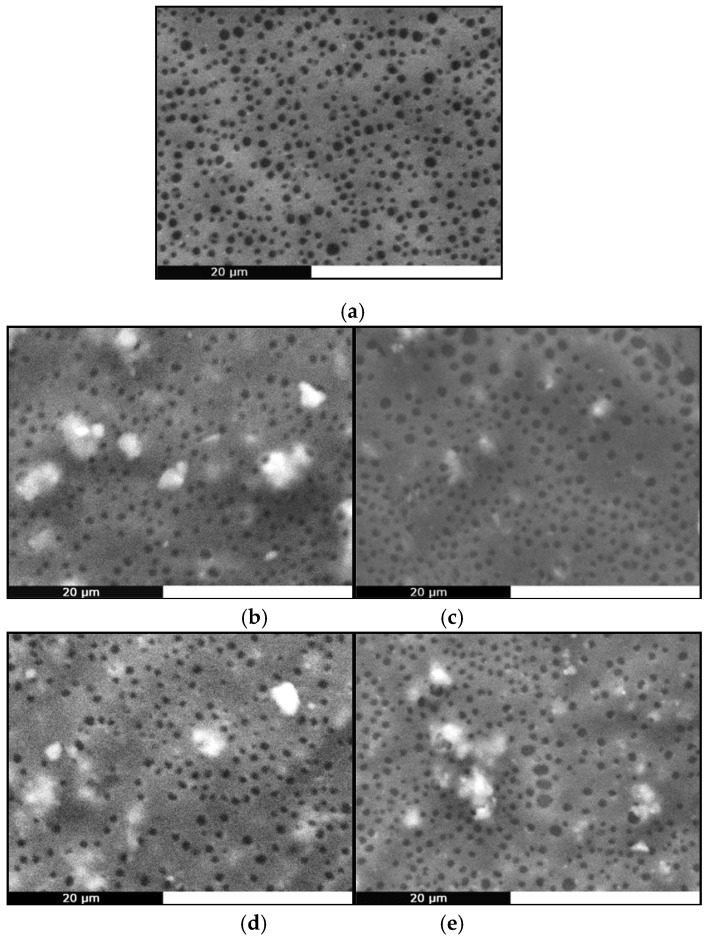
SEM micrographs of (**a**) PCL, (**b**) PCL/CaBG, (**c**) PCL/SrBG, (**d**) PCL/CaBG-Iba, (**e**) PCL/SrBG-Iba.

SEM analysis (Figure 9) showed that all membranes had a porous surface, most likely due to the fast evaporation of the solvent during spin coating [28]. This is a highly desirable feature for any material intended for tissue engineering applications, since surface porosity helps the adhesion and proliferation of cells. The pore size is smaller than the usual size of cells (10 μm) so the prepared films could be used as occlusion membranes. In addition, all composite membranes exhibit a coarser structure than the smooth neat PCL film, due to the presence of the additives, which have also formed some aggregates. Bioglass^®^ particles have been found capable of providing otherwise smooth polymeric films with surface irregularities [5]. Typically, spin coating of PCL solutions in chloroform gives smooth, flat and non-porous membranes [64,98,99], suggesting the final morphology of the film depends on the solvent used as well as on the subsequent thermal and/or vacuum treatment. Dziadek et al. recently confirmed this hypothesis, when they found that different solvents interact differently with PCL, creating solutions with unique features that result in polymeric films with controlled topography, crystallinity, mechanical properties and wettability [100].

#### 2.2.2. Structural Characterization

The chemical structure of the prepared films was studied by FTIR spectroscopy (Figure S2). All peaks are attributed to stretching vibrations of the C–H, C=O and C–O bonds [63,101]. The absence of the hydroxyl peak is characteristic and is due to the high molecular weight of the synthesized PCL. The spectra of all composite membranes depict an additional small peak at 452 cm^−1^ associated with the vibrations of the Si–O–Si and O–Si–O bonds of the BGs. Otherwise, the absence of new peaks and/or shifts indicates the absence of strong interactions between the PCL matrix and the fillers [102].

#### 2.2.3. Thermal Properties

Table 2 shows the melting point, T_m_, crystallization temperature, T_c_, and crystallinity, X_c_ values of PCL, Iba, and the respective composite films, as derived from DSC analysis. The respective DSC thermograms are presented in Figure S3. Iba is a polymorphic drug and this may explain the occurrence of two melting peaks, at 142 °C and 180 °C.

The neat PCL membrane has a T_m_ = 58.3 °C and a crystallinity of 54.2%, while the respective values for the bulk material are 65.4 °C and 60% [63], indicating that the spin coating processing has a significant impact on the thermal properties of PCL. This effect may be directly related to the dissolution process and solvent evaporation, which even in very small quantities can act as a kind of plasticizer for the polymer, resulting to a lower T_m_ and crystallinity. The composite membranes had quite similar T_m_ and T_c_ values with PCL. It should be noted that the thermograms of the PCL/CaBG-Iba and PCL/SrBG-Iba membranes did not show any drug-related peaks, which may indicate either its presence in an amorphous state, or its complete confinement in the pores of the BGs. The degree of crystallinity, however, decreased up to 22.5% in the presence of the bioactive glasses.

Addition of bioactive glass particles with sizes larger than 3 μm has been reported to decrease the T_m_ and X_c_ of PCL and this reduction has been attributed to the formation of defective crystals and hindering of macromolecular chain mobility [100,103], while on the contrary BGs with diameter <1 μm seem to have a beneficial effect on the thermal properties and crystallinity of the membrane. Another important factor that has great impact on thermal properties are the interfacial interactions between the polymeric matrix and the filler [104]. In a recent work of our group it was concluded that the synthesis of PCL nanocomposites with different nanosized mesoporous BGs resulted in strong interfacial interactions between the polymer and the fillers which had a great impact on improvement of thermal properties [63]. Thermal stability of the films was studied by TGA (Figure S4). PCL is a fairly thermally stable polymer that degrades in one step at a T_max_ of 420 °C. This is shifted to lower temperatures by about 10 °C in the composite PCL/bioglass films, indicating their lower thermal stability. According to literature, bioglass particles dramatically reduce the thermal stability of biodegradable polymers, because of the hydroxyl groups on the particle surface. These groups, in the presence of moisture, can be linked to the Ca^2+^ counter-ion catalyzing chain cleavage effects [105]. Nevertheless, the PCL/CaBG-Iba and PCL/SrBG-Iba films have slightly better thermal stability than those without Iba.

#### 2.2.4. Mechanical Properties

The loading-unloading indentation curves of neat PCL, PCL/SrBG and PCL/CaBG as well as those loaded with Iba (PCL/SrBG-Iba and PCL/CaBG-Iba) are shown in Figure S5. The indentation force-depth curves for all materials under test specified a creep phenomenon at the peak force of 10 mN. PCL has shown larger creep than the composite membranes, while all loading and unloading curves have shown no discontinuities or steps, indicating that no cracks were formed during indentation. The indentation depths at the peak load range approximately between 1.7–2.2 μm for the PCL composites and around 3.2 μm for the neat PCL. The plastic work done was significantly higher for PCL, as revealed from the increased area enclosed between the loading and unloading curve. On the contrary, that softening behavior was not observed in the loading-unloading response of all composite membranes. In addition, the PCL/SrBG-Iba has shown the lowest indentation depth as compared with all other materials under study.

**Figure 10:**
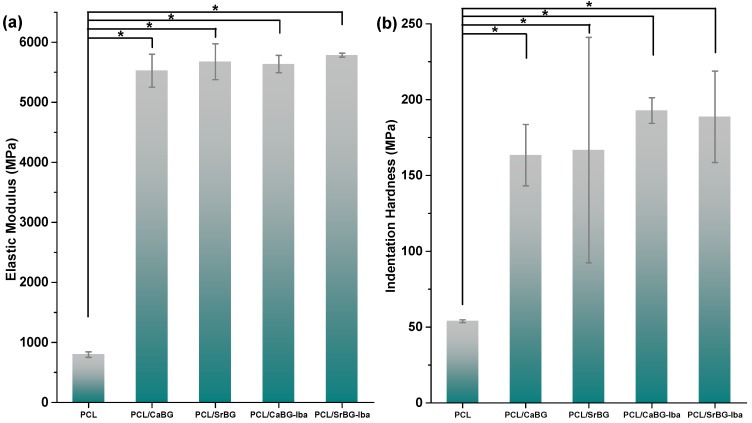
(**a**) Elastic Modulus and (**b**) Hardness of PCL membranes. *: *p* < 0.05.

Figure 10 shows the results from the indentation test. The modulus of neat PCL was 797 MPa, while the elastic modulus of PCL/SrBG-Iba was the highest obtained as compared to the results from the other materials under study with a value of 5784 MPa. The indentation hardness measurements followed the same pattern, with a small hardness increase by the addition of Iba with the highest values obtained for PCL/CaBG-Iba. As expected, the addition of BGs in the PCL films resulted in a statistically significant increase in the indentation hardness as well as in the elastic modulus. The differences between the composite membranes were insignificant.

#### 2.2.5. Hydrophilicity

One of the most important parameters for enabling adequate adhesion and cell proliferation on the surface of a material is its hydrophilicity. PCL, although biodegradable and bioresorbable, is hydrophobic and as a result it degrades slowly. A simple and effective method for increasing the hydrophilicity of PCL is the introduction of hydrophilic particles such as BGs. BG particles have been reported to increase the hydrophilicity and water sorption properties of PCL, allowing the tailoring of the degradation rate of the polymer in physiological environment [106].

**Figure 11:**
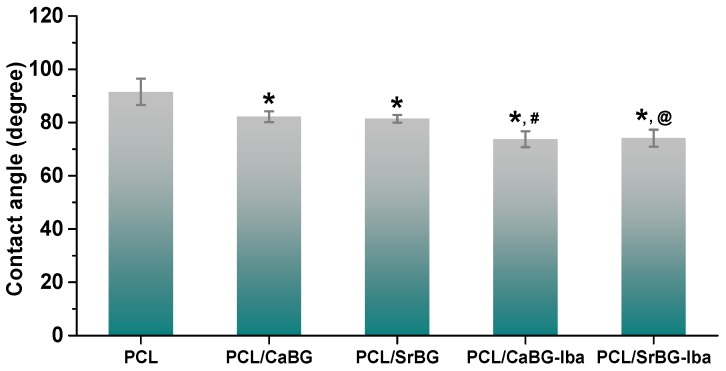
Water contact angle values of composite thin films. *: *p* < 0.05 compared to PCL, # compared to PCL/CaBG, @ compared to PCL/SrBG.

Water contact angle measurements showed a contact angle of 91.5° for neat PCL (Figure 11). After the introduction of CaBG and SrBG particles this value drops to about 82°, due to the hydrophilic character of the two additives [106]. The contact angle decreases even further, and the membranes become even more hydrophilic after the incorporation of Iba loaded BGs, which was expected since the drug is freely soluble in water.

#### 2.2.6. In Vitro Bioactivity

Apatite deposition is necessary for the formation of a bioactive interface between biomaterials and tissues, since it can promote the proliferation and differentiation of osteoblasts [48]. Bioactivity is one of the most attractive features of BGs, since their incorporation in bioinert polymeric matrices can impart on them significant bioactivity [106,107].

**Figure 12:**
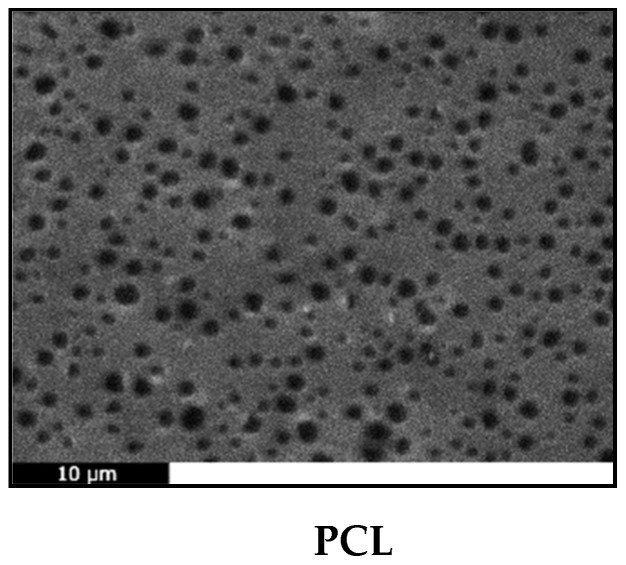

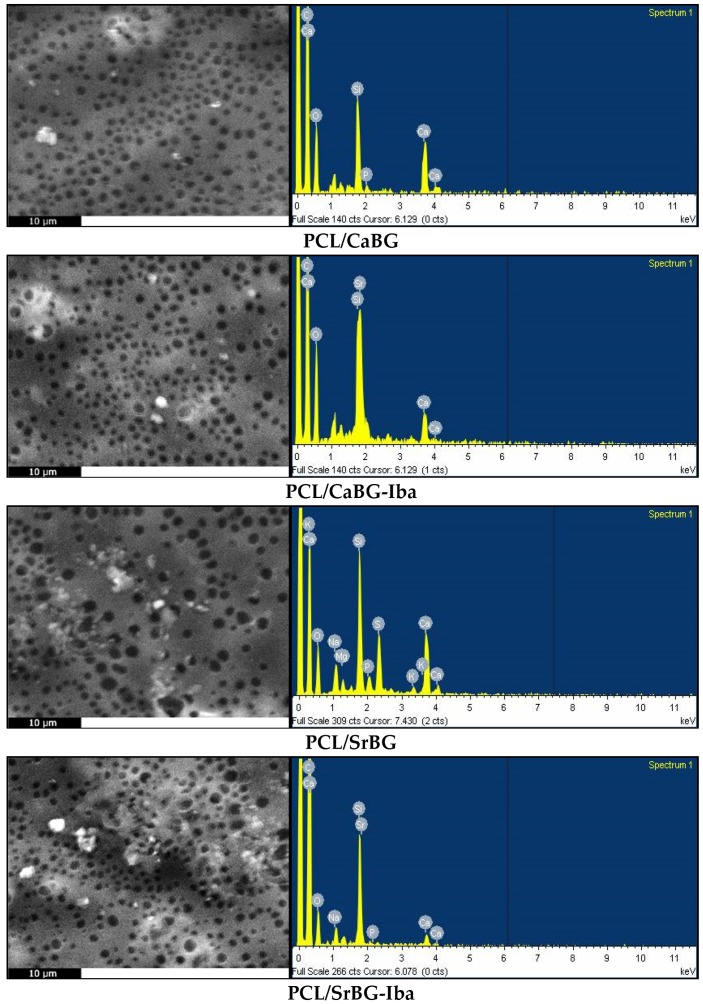
SEM images and EDX spectra of PCL and its composite thins films before and after 14 days of soaking in SBF.

In vitro bioactivity of the PCL membranes was investigated by immersing them in SBF for 14 days followed by observation of their surface by SEM and EDX spectra (Figure 12). As expected, no hydroxyapatite was formed on the surface of neat PCL, which is considered a bioinert material. In contrast, scattered spherical particles with a composition similar to that expected for hydroxyapatite were observed on the surface of the composite films. The addition of SrBG seems to enhance more the bioactivity of PCL than CaBG, in agreement with the bioactivity testing of the standalone bioglasses (Figure 5).

The number of these particles increases in the presence of Iba for both PCL/CaBG-Iba and PCL/SrBG-Iba membranes, suggesting that Iba further enhances the bioactivity of PCL membranes.

#### 2.2.7. Ion Release

**Figure 13:**
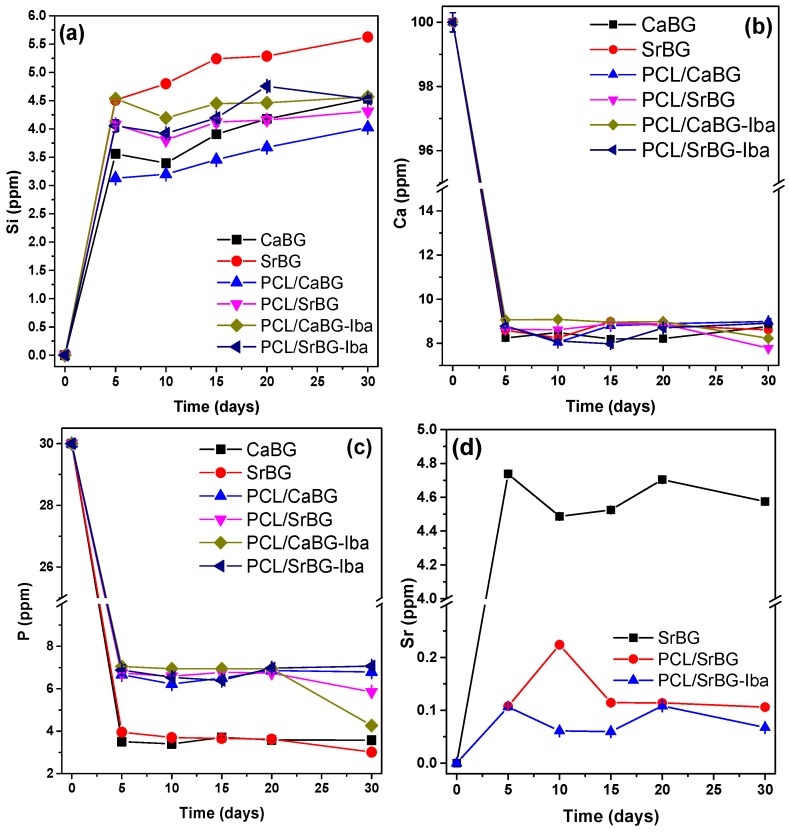
Release of (**a**) Si, (**b**) Ca, (**c**) P and (**d**) Sr ions from composite PCL films and pristine CaBG and SrBG BGs.

Testing of bioactivity in SBF provides with useful preliminary results, but ionic dissolution products and their concentration are also essential for stimulating the growth and differentiation of osteoblasts [108]. Therefore, the amounts of Si, Ca, P and Sr released when the BGs and the PCL membranes were exposed to SBF for 30 days were quantified with ICP-MS. The results are shown in Figure 13. The respective measurements showed an increasing release of Si ions (Figure 13a) with time for all samples (pristine BGs and composite films). Changes in the concentrations of ions during incubation of BG containing samples in SBF is a result of both dissolution and precipitation. Initially, ion concentrations are expected to increase due to dissolution of the BGs, followed by precipitation of apatite or amorphous calcium phosphate that cause their consumption from the solution [109,110].

Concentration of Si increased continuously with immersion duration (Figure 13a). When in contact with SBF, cation exchange between the buffer solution and the BGs takes place. This cation exchange results in the formation of Si–OH groups that increase the pH, which in turn results in cleavage of Si–O–Si bonds, therefore releasing Si ions in the solution. The amount as well as the release rate of Si was higher for the samples with Sr-substituted BG compared to CaBG, as the BGs with Sr have a more expanded network and thus are more soluble in aqueous solutions [111,112].

A large amount of P and Ca ions is consumed during the first 5 days of immersion of the samples in SBF, as shown in Figure 13b,c. During biomineralization of the surface of bioactive materials, a CaO-P_2_O_5_ layer is formed after the migration of PO_4_^3−^ and Ca^2+^ ions from SBF [43], resulting in decrease of the concentration of P and Ca. The consumption of both Ca and P from CaBG and SrBG occurred at an almost identical extend suggesting their bioactivity is very similar, which is in agreement with the FTIR and XRD measurements of Figure 5. These results highlight the fact that the total substitution of Ca with Sr does not negatively affect the bioactivity of the BG.

In all cases, biomineralization seems to have taken place already during the first 5 days of incubation for both the BG powders as well as the membranes. After the 20th day, a new reduction in the concentration of Ca and P ions is observed for most samples which is indicative of the formation of amorphous calcium phosphate (ACP) during the 4^th^ step of hydroxyapatite formation [113].

In the case of Sr, the amounts released are relatively stable with time indicating rapid initial release. The concentration of Sr^2+^ released from SrBG is approximately 4.5 ppm, while the SrBG containing membranes released Sr ions in the range of ppb. It is well known that the beneficial effect of Sr ions on osteoblasts is dose dependent. It has been reported that Sr^2+^ concentrations from 8.7 to 87.6 ppm have a stimulatory effect on osteoblasts in vitro [114], while larger amounts result to apoptosis [115].

#### 2.2.8. In Vitro Cytocompatibility and Osteogenic Potential

**Figure 14:**
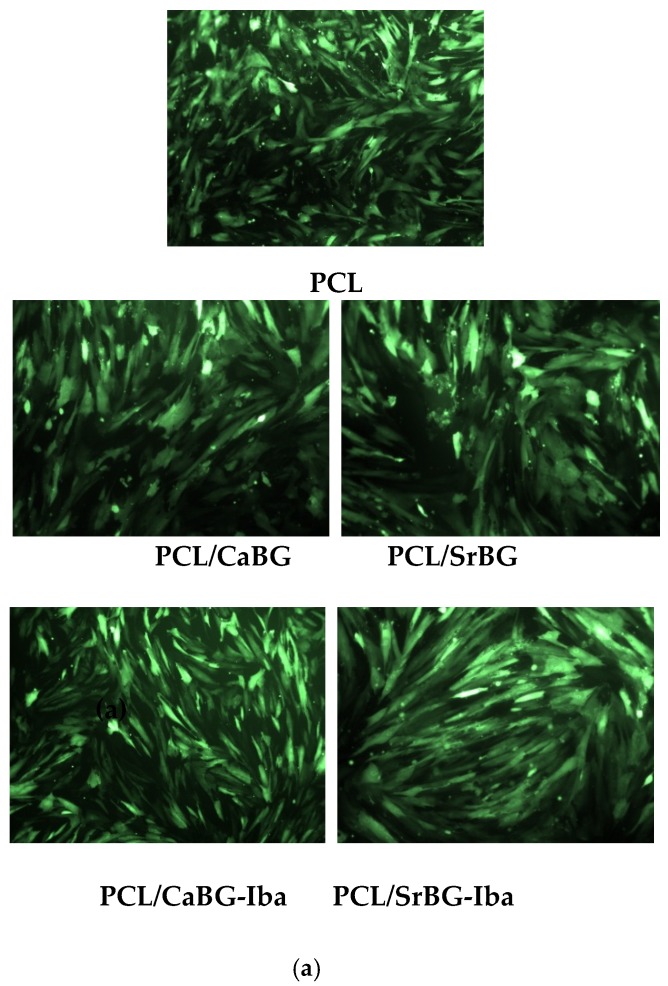

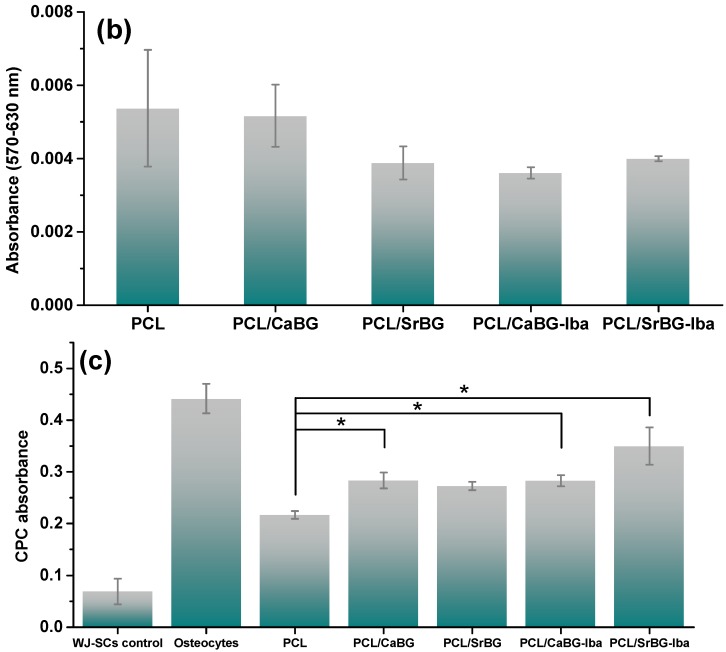
(**a**) Fluorescence microscopy images of genetically modified WJ-MSCs on the PCL membranes, (**b**) MTT assay results of WJ-MSCs cells after seeding for 24 h on PCL and its nanocomposites and (**c**) assessment of osteogenic differentiation by cetylpiridinium chloride (CPC) method after 28 days in the induced-differentiation culture 30 days culture. *: *p* < 0.05.

WJ-MSCs, easily isolated from cord tissue during birth, consist one of the most common stem cell sources for induced differentiation assays. The biocompatibility of PCL and its composite films incorporating CaBG/CaBG-Iba and SrBG/SrBG-Iba was evaluated by seeding WJ-MSCs on the surface of the materials. Cell viability was assessed after 24 h with fluorescence microscopy, as presented in Figure 14a.

In all cases, no cytotoxicity was observed after 24 h. The viable cells appear as bright green spots that adhered and grew on all films. Fluorescence microscopy findings were further supported by MTT assay results (Figure 14b). The performed t-test proved that there are no significant differences in the metabolic activity of the films, thus demonstrating the viability of the cells. None of the films appeared to cause cytotoxicity (since the Sr content in the PCL/ SrBG and PCL/SrBG-Iba films was too small—in the ppb range as already shown in Figure 13—to have any cytotoxic effect). This is confirmed by the fluorescence micrographs, with the cells adhering and multiplying on the film’s surfaces, indicating their biocompatibility.

The osteogenic differentiation of WJ-MSCs to osteocytes on PCL and its nanocomposites was evaluated using the cetylpiridinium chloride (CPC) method after 30 days of induced cell culture towards osteocytes [116]. As shown in Figure 14c, WJ-MSCs attached to the PCL/SrBG-Iba sample presented the highest differentiation potential to osteocytes in comparison with neat PCL, which could possibly be attributed to the synergistic effect of Sr and Iba on osteogenesis. It is noteworthy that the cellular attachment on PCL, instead of the plastic surface of the culture plates, reduces their effective differentiation to osteocytes as evidenced by the stronger differentiation of the control group WJ-SCs (negative control) to osteocytes (positive control). However, the incorporation of osteoinductive additives in PCL matrices seems to facilitate the osteoinductive medium mediated-differentiation statistically significant compared to the PCL group.

Recently, PCL-based scaffolds containing osteoinductive molecules were developed for accelerated or enhanced osteogenic differentiation and bone tissue regeneration [58]. On this basis, the incorporation of Sr-containing BGs in combination with bisphosphonates in PCL seems to be an effective strategy to modulate its osteogenic potential and possibly stimulate osteogenic differentiation. The incorporation of osteoinductive additives in PCL matrices could promote bone tissue formation by avoiding the introduction of osteoinductive mediums in cell cultures, with the goal of minimizing the addition of exogenous growth factors. The use of non-cytotoxic materials as mediators of induced differentiation towards a desirable tissue could widen the applications of biomaterials in regenerative medicine.

## 3. Materials and Methods

### 3.1. Materials

ε-Caprolactone (CL) monomer (purity 99%), tin(II) 2-ethylhexanoate (TEH) catalyst (analytical grade), poly(ethylene glycol) (PEG) (average Mn 10.000 g/mol), tetraethyl orthosilicate (TEOS) (reagent grade 98%), triethyl phosphate (TEP) (purum 99.8+%), calcium nitrate tetrahydrate Ca(NO_3_)_2_·4H_2_O) (ACS reagent 99%), Sr(NO_3_)_2_ (ACS reagent, ≥99%), cetyltrimethylammonium bromide (CTAB) and ethanol were purchased from Sigma Aldrich Chemical Company (Saint Louis, MO, USA). Sodium hydrate pellets were supplied from Mallinckrodt Company (Staines-upon-Thames, UK). Ibandronate API (Iba) was kindly donated by Pharmathen SA (Athens, Greece).

### 3.2. Synthesis of Mesoporous Ternary Bioglasses

Two different types of mesoporous ternary BGs (SiO_2_-CaO/SrO-P_2_O_5_) were prepared through a hydrothermal method based on a previously described protocol [63], using PEG and CTAB, as non-ionic co-surfactant and cationic surfactant, respectively. CaO was fully replaced by Sr (10% on molar ratio). Briefly, for the preparation of the ternary bioglass with 80% SiO_2_, 10% SrO, 10% P_2_O_5_ molar ratio (hereafter denoted as SrBG), proper amounts of PEG and sodium hydrate were dissolved in distilled H_2_O under vigorous stirring, followed by the addition of CTAB. After stirring for 1 h at room temperature, Sr(NO_3_)_2_, TEOS and TEP were added to the mixture, which was kept at room temperature under vigorous stirring for 24 h and then transferred into Teflon-lined autoclaves. The autoclaves were sealed and heated at 80 °C for 48 h and then allowed to cool down to room temperature. The reaction products were obtained by filtration after washing with H_2_O and ethanol and drying at 100 °C overnight. Finally, the white powder obtained was calcined in air at 600 °C for 5 h with a heating rate of 9 °C/min. The same protocol, with the necessary adaptations, was also followed to obtain the Ca-containing bioglass systems (hereafter denoted as CaBG), in which Ca (NO_3_)_2_·4H_2_O precursor was used as a calcium source.

### 3.3. Synthesis of PCL via Ring Opening Polymerization (ROP)

PCL was synthesized with the ring opening polymerization (ROP) of ε-caprolactone (ε-CL) monomer, based on a previously described protocol [63]. More specifically, ε-CL was dried over CaH_2_ and purified by distillation under reduced pressure prior to use. The bulk polymerization of ε-CL was carried out in a 250 mL round-bottom flask equipped with a mechanical stirrer and a vacuum apparatus. The catalyst TEH was added as a solution in toluene at a final concentration of 1 × 10^−4^ mol per mol of monomer and the mixture was degassed and purged with dry Ar. The reaction was carried out for 3 h at 190 °C, followed by increasing the reaction temperature from 210 to 240 °C over a period of 90 min. Monomers were removed through distillation by gradually applying high vacuum (≈5 Pa), to avoid excessive foaming. Polymerization was terminated by rapid cooling to room temperature. Molecular weight per weight, as measured by gel permeation chromatography, had a value of 71900 g/mol and a polydispersity index of 1.49 [63].

### 3.4. Loading of CaBG and SrBG Bioglasses with Ibandronate

CaBG and SrBG were loaded with Iba (denoted as CaBG-Iba and SrBG-Iba, respectively), through the following procedure. 500 mg of each bioglass was dispersed in a 0.5 wt% aqueous solution of the drug under magnetic stirring for 3 days. Each dispersion was finally centrifuged at 4000 rpm and the obtained solid was dried under vacuum.

### 3.5. Preparation of PCL Composite Thin Films with Bioglasses

Both bare and drug loaded bioglass particles were combined with PCL for the preparation of composite thin films by spin coating (Model WS-650MZ-23NPPBO, Laurell Technologies Corporation, North Wales, PA, USA). Before the experiments, glass slides with dimensions 2 × 2 cm and thickness 0.13–0.17 mm were cleaned with acetone, rinsed with deionized water and dried under a nitrogen purge. After systematic spin coating trials using a 10 wt% PCL solution in dichloromethane, the optimum rotation speed of 6000 rpm and a volume of 0.6–0.7 mL for each sample was used. These conditions were employed for the preparation of the composite films, using as feed a dispersion of both BGs (with and without Iba) in the PCL solution at a concentration of 10 wt% (with respect to the total PCL mass). The fillers were dispersed in the solution with an ultrasonic probe. A continuous flow of nitrogen and vacuum were used during processing, which resulted in four composite materials, i.e., the drug-free PCL/CaBG, PCL/SrBG and those incorporating Iba, PCL/CaBG-Iba and PCL/SrBG-Iba. Neat PCL thin films were also prepared and used as reference. After the spinning process, the coated glass slides were stored in a fume hood while allowing the solvent to evaporate for 24 h and were finally dried under vacuum oven at 30 °C for 24 h to remove any residual solvent.

### 3.6. Characterization

#### 3.6.1. Characterization of CaBG and SrBG

The morphological features of the BGs were assessed through a Phenom ProX (Phenol-World BV, Eindhoven, The Netherlands) scanning electron microscope (SEM) equipped with an energy-dispersive X-ray. The samples were attached on a metal stub with double-sided carbon tape (Ted Pella Inc, Redding, CA, USA) and inserted in the scanning electron microscopy’s vacuum chamber using a special charge reduction sample holder. This sample holder is designed to reduce sample charging and eliminate extra sample preparation of non-conductive samples. The morphology of the membranes was examined with a JMS-840A SEM instrument (JEOL, Akishima, Japan) equipped with an energy-dispersive X-ray Oxford ISIS 300 microanalytical system (Oxford Instruments, Tubney Woods, Abingdon, UK). All samples were coated with carbon black to avoid charging under the electron beam.

Particle size distribution of the prepared nanoparticles was determined by dynamic light scattering (DLS) using a Zetasizer Nano instrument (ZEN 3600; Malvern Instruments, Malvern, Worcestershire, UK) operating with a 532 nm laser. A suitable amount of each BG was dispersed in distilled water to a final concentration of 1‰ and was ultrasonicated before the measurement. For each sample, three measurements were conducted.

Fourier transform infrared spectroscopy (FTIR) spectra of the BGs before and after Iba adsorption were obtained using a model Spectrum One FTIR spectrometer (Perkin-Elmer, Waltham MA, USA).

The powder X-ray diffraction (XRD) patterns of the BGs were recorded on a Rigaku R-AXIS IV Imaging Plate Detector mounted on a Rigaku RU-H3R Rotating Copper Anode X-ray Generator (λ = 0.154 nm). The XRD patterns of Iba and of the BGs after Iba adsorption were were obtained using a MiniFlex II XRD system from Rigaku Co. (Tokyo, Japan), with CuKα radiation (λ = 0.154 nm) in the angle 2θ range from 5 to 80°.

The pore properties of the BGs were determined by the nitrogen adsorption/desorption measurements at 77 K using a volumetric gas adsorption analyser (AUTOSORB-1-MP, Quantachrome Instruments, Boynton Beach, FL, USA). Prior to measurement, the samples were appropriately outgassed (at 250 °C for 12 h) under ultra-high vacuum (10^−6^ mbar), while ultra-pure N_2_ was used. The BET area values were calculated by the Brunauer-Emmett-Teller (BET) method, following the BET consistency criteria. Pore size distributions were deduced by fitting the adsorption isotherms based on a non-local density functional theory (NLDFT) kernel developed for N_2_ at 77 K on silica materials with cylindrical pores.

Thermogravimetric analysis, before and after the adsorption of Iba, was carried out with a Setsys16/18 TG-DTA system (Setaram Instrumentation, Caluire, France). Samples were placed in alumina crucibles and heated from ambient temperature to 800 °C at 20 °C/min using a 50 mL/min flow of N_2_; an empty alumina crucible was used as reference.

The in vitro bioactivity of the BGs was assessed by monitoring the formation of an apatite layer on the surface of the synthesized glasses that were immersed in a simulated body fluid (SBF) solution buffered at pH 7.4 [117]. The samples were soaked in SBF in a concentration of 1.5 mg/mL and incubated in closed falcon tubes at 37 °C for 1, 3, 7 and 14 days. The falcon tubes were placed inside a shaking incubator (LSI-3016R, Labtech, Heathfield, United Kingdom) at a fixed temperature of 37 °C and speed 200 rpm. At the selected time points, the powders were separated from the SBF solution by centrifugation at 5000 rpm for 5 min, rinsed with acetone to cease further reactions, air dried at room temperature and analyzed using FTIR and XRD (Rigaku R-Axis IV) to detect the HA phase formation.

#### 3.6.2. Characterization of the Composite Films

The morphology of the PCL composite films was examined using a JEOL JMS-840A scanning electron microscope (SEM) equipped with an energy-dispersive X-ray Oxford ISIS 300 microanalytical system (Oxford Instruments). All samples were coated with carbon black to avoid charging under the electron beam.

FTIR spectra of the thin films were obtained using a Perkin-Elmer FTIR spectrometer (Waltham MA, USA), model Spectrum One in absorbance mode and in the spectral region of 400–4.000 cm^−1^ using a resolution of 4 cm^−1^ and 64 co-added scans.

Differential scanning calorimetry (DSC) measurements were performed with a Pyris-6 instrument (Perkin Elmer, Waltham, MA, USA) calibrated with Indium and Zinc standards, under N_2_ flow. For each measurement, 5–10 mg of each sample was placed in a sealed aluminum pan and heated from ambient temperature to 100 °C with a heating rate of 10 °C/min, and subsequently cooled to 10 °C with a cooling rate of 5 °C/min. The degree of crystallinity X_c_ (%) was calculated with the equation:

X_c_ = ΔH_m_/(1 − x)ΔH^0^_m_% (1)

where ΔH_m_ is the melting enthalpy, x is the weight fraction of the BG particles and ΔH^0^_m_ the theoretical heat of fusion for 100% crystalline PCL, 135 J/g [118].

Thermogravimetric analysis was carried out with a Setsys16/18 TG-DTA system (Setaram Instrumentation, Caluire, France). Samples were placed in alumina crucibles and heated from ambient temperature to 620 °C at 20 °C/min using a 50 mL/min flow of N_2_; an empty alumina crucible was used as reference.

The materials under study were assessed through nanoindentation tests in order to compare their modulus and hardness. In instrumented indentation tests the load is measured as a function of penetration depth. Such tests enable local variations of modulus and hardness to be measured precisely [119,120,121,122]. In the current work the indentations were conducted using a dynamic ultra-micro-hardness tester (DUH-211; Shimadzu Co., Kyoto, Japan) fitted with a triangular pyramid indenter tip (Berkovich indenter). The indentations made on the surface of the nanocomposites under study appeared as an equilateral triangle. Ten measurements were conducted on each specimen, which were purposely scattered on the surface. After contact of the indenter with the surface, this was driven into the surface until a peak load of 10 mN was reached. The peak load was held for 3 s (in order to minimize the effect of viscoelastic deformation of the specimen, notably creep, on property measurements) and then the indenter was unloaded, to a load of zero. The statistical significance was analyzed by Student’s t-test. A *p*-value < 0.05 was considered as statistically significant.

The apparent contact angle of water was measured on a contact angle analyzer (Kruss EasyDrop Standard, Hamburg, Germany), by gently placing a water droplet (5 µL) on the surface of the polymeric films. The presented values were averaged over at least three points for each sample, using the circle fitting method. The statistical significance was analyzed by Student’s t-test. A *p*-value < 0.05 was considered as statistically significant.

For the in vitro biomineralization experiments, the nanocomposites in the form of films were soaked in SBF, buffered at pH 7.4, and incubated at 37 °C in closed containers for 14 days. After the immersion test, the films were removed and washed three times with deionized water to remove adsorbed minerals. The films were dried under vacuum and characterized by SEM using the conditions described previously.

An iCAP Q ICP-MS (Thermo Scientific, Fisher Scientific, Ottawa, ON, Canada) equipped with a PFA-ST nebulizer, nickel interface cones and 2.5 mm quartz injector with Qtegra™ Intelligent Scientific Data Solution™ Software was used for the determination of ion concentration (Si, Ca, P, Sr) after soaking of the BGs as well as the composite films in SBF for 30 days. The concentration of the samples in SBF was 60 mg/mL for the BGs and 0.04 mg/mL for the membranes. The concentrations of trace elements were measured by ICP-MS with ‘in-sample switching’ between two operational modes: standard mode and kinetic energy discrimination with He as the cell gas to reduce polyatomic interferences. Internal standard included Ir (10 μg L−1) in 2% trace analytical grade (TAG) HNO_3_. Multi-element solutions as well as single element solutions were diluted as appropriate to create the calibration curves with concentrations of 0.100, 1.00, 2.00, 5.00, 10.0, 25.0, 50.0 and 100 μg/L. A linear regression was confirmed for all selected elements (R^2^ > 0.999). Using ^193^Ir as the internal standard, all samples were analyzed in triplicate, with instrument parameters of 10 sweeps and 10 ms dwell time for all elements. For QA/QC, blanks (2% HNO_3_) and Reference Material (Proficiency testing, SCHEMA) were analyzed immediately after calibrating the instrument and after every 10 samples. All the blank readings were lower than the instrument detection limits, and the element concentrations determined in the reference material were within 10% of the certified values.

### 3.7. Cell Studies

#### 3.7.1. Isolation, Cultivation and Genetic Modification of Wharton’s Jelly-Derived Mesenchymal Stem Cells (WJ-MSCs)

Thirty five mL of umbilical cord blood were collected with the parents’ informed consent in a sterilized box. After a saline wash and a mild cut up with a lancet, an overnight lysis with 4 mg/mL collagenase, 2 mg/mL dispase in PBS was performed in a stirring incubator. The next day the mixture was filtered through a 70 μm Filter Unit and was subsequently centrifuged at 850 g for 10 min at room temperature (RT). The obtained pellet was resuspended in DMEM medium supplemented with 10% fetal bovine serum (FBS) and 1% penicillin/streptomycin (DMEM full medium) and was then incubated in culture flasks for 72 h in a 37 °C incubator with 5% CO_2_. Between the 4th and the 5th passage we performed a Pt2-Venus-neo mediated nucleofection. Approximately 4 × 10^5^ cells were mixed with 7.5 μg of plasmid DNA SB100X transposase and pT2-Venus-neo transposon expression plasmids (1:10 ratio) and a subsequent electroporation was performed according to the manufacturer’s protocol (Lonzabio, Basel. Switzerland). The cells were then incubated in a well of a 6-well plate in the presence of DMEM full medium until a 90% confluency after which 100 mg/mL G418 was added for the selection of the genetically modified WJ-MSCs.

#### 3.7.2. Sterilization of the Materials and WJ-SCs Plating

All PCL films were cut into 5 × 5 mm pieces and sterilized in gradually reduced ethanol concentrations (100–70–50%). Next, they were washed three times with ddH_2_O and air dried for 4 h under sterile conditions. Fibrin glue was prepared after blood sampling of a healthy volunteer donor. 15 μL of fibrin glue per film were placed in the bottom of a 24-well plate and the materials were seeded from the top using a sterile pincher by applying minimal manual pressure and air dried overnight under sterile conditions. WJ-SCs were detached using Trypsin-EDTA 1x in PBS and were counted in a Neubauer cell counting chamber. 5 × 10^4^ cells/well of each condition were resuspended in DMEM full medium and were subsequently placed above the films. Upon air drying for 4 h in the incubator, 1 ml DMEM full medium was added per well for the culture initiation either for MTT assay or for osteoinductive differentiation procedure as described below. For the cytotoxicity measurements that performed to CaBG and SrBG samples, NPs were added in the culture supernatants at two different concentrations: 100 and 200 ug/mL in DMEM. For the sterilization of the solutions, 0.22 μm filter units were used. Non-NP treated cells were used as a control group in the same number as the other groups.

#### 3.7.3. 3-[4,5-Dimethylthiazole-2-yl]-2,5-diphenyltetrazolium Bromide (MTT) Assay

In order to assess the cytotoxicity levels of materials, the MTT assay was performed (Sigma-Aldrich, Saint Louis, MO, USA) 24 h after the initial cell plating. Briefly, after the medium removal from the wells, MTT reactant was introduced in a ratio of 1:10 in DMEM culture medium and was followed by a 4 h incubation in 37 °C with 5% CO_2_. Upon the removal of the MTT, 1 mL/well of DMSO was introduced for one additional hour of incubation in the same conditions. The reduction of MTT was counted at wavelengths 570 and 630 nm (Perkin Elmer).

#### 3.7.4. Observation under Fluorescence Microscope

The observation of the cells above the materials was performed under a fluorescence HBO 50 mercury lamp as well as reflectors with fluorescence filter (excitation 488 nm, emission 509 nm), while the program for downloading and editing the photos was the Fluorescence Lite software module of AxioVision LE (Carl Zeiss, Oberkochen, Germany).

#### 3.7.5. Osteogenic Differentiation—Alizarin Red Staining and CPC Quantification

The induction of differentiation towards bone cells was accomplished by introducing osteogenesis medium, PT-3002 hMSC Osteogenic Differentiation BulletKit (Lonza, Switzerland), in the culture for 28 days. Cells plated in plastic surfaces were used as a control group, in the same number as the rest of the other groups. The successful induction of differentiation towards bone cells was verified with Alizarin Red staining. The quantification of the procedure was carried out with 10% cetylpyridinium chloride (CPC) in 10 nM sodium phosphate, pH 7.0 for 15 min in RT. Τhe extracts were 10 times dissolved in 10% CPC and the Alizarin Red concentration was counted at 562 nm in a Perkin Elmer spectrometer (Waltham MA, USA).

#### 3.7.6. Statistical Analysis

All cell experiments were performed in triplicate and the results were expressed as mean ± standard deviation. The statistical significance was analyzed by Student’s t-test. A *p*-value < 0.05 was considered as statistically significant.

#### 3.7.7. Ethical Statement

This study was approved by the Ethics Committee of Aristotle University of Thessaloniki School of Medicine (390-9/1.7.2017). This research involves Human Participants after their informed consent. Peripheral blood samples were collected from healthy volunteer donors while mesenchymal stromal cells were isolated from Wharton’s jelly after parent’s approval during stem cell banking in Biohellenika SA Biotechnology Company (Thessaloniki, Greece).

## 4. Conclusions

Two different mesoporous, sub-micron bioactive glasses with different compositions and large surface areas were prepared with a hydrothermal method. The bioglasses were loaded with ibandronate and used in the preparation of composite thin membranes of poly (e-caprolactone) by the method of spin coating for GBR/GTR applications. The presence of both BGs affected positively the mechanical properties of PCL and its hydrophilicity. The incorporation of Sr^2+^ ions induces significant bioactivity, which is further enhanced in the presence of ibandronate. All membranes were found to be biocompatible after seeding WJ-MSCs on their surface. WJ-MSCs cultured on PCL-SrBG-Iba sample presented a significant potential for differentiation to osteocytes, suggesting a possible synergistic effect of Sr and Iba on osteogenesis.

Potential limitations of this study include the inability to quantify the in vitro drug release rate due to its low content and the use of a single method for the estimation of osteogenic differentiation. Further studies are needed to confirm the osteogenic potential of the prepared membranes, quantification of the drug’s release rate in different media and finally conducting of in vivo tests to determine if these membranes can be introduced to clinical practice in the future.

## Figures and Tables

**Table 1:** EDX elemental analysis of CaBG and SrBG (% atomic and weight concentrations, n = 3).

	CaBG		SrBG	
Element	Atomic conc. (%)	Weight conc. (%)	Atomic conc. (%)	Weight conc. (%)
O	64.39 ± 2.87	49.58 ± 2.98	57.89 ± 2.01	38.59 ± 2.10
Si	31.39 ± 2.75	42.33 ± 3.03	37.06 ± 1.65	43.30 ± 1.11
P	0.10 ± 0.06	0.14 ± 0.09	0.13 ± 0.02	0.17 ± 0.04
Ca	4.13 ± 0.19	7.94 ± 0.30	0	0
Sr	0	0	4.92 ± 0.38	17.94 ± 1.06

**Table 2:** Thermal properties of PCL and its composite thin films.

Sample	Tm (°C)	DHm (J/g)	Tc (°C)	DHc (J/g)	Xc (%)
Iba	142.4, 179.7	173.64, 25.90	−	−	
PCL	58.3	73.16	34.7	−57.96	54.2
PCL/CaBG	57.5	53.81	35.1	−47.40	35.9
PCL/SrBG	58.5	50.83	34.6	−43.04	33.9
PCL/CaBG-Iba	59.2	47.60	34.8	−39.81	31.7
PCL/SrBG-Iba	59.4	54.75	34.1	−51.06	36.5

## Supplementary Materials

The following are available online. Figure S1: DSC thermograms of CaBG-Iba and SrBG-Iba during heating with 20 °C/min, Figure S2: FT-IR spectra of the composite membranes, Figure S3: DSC thermograms of (a) Iba upon heating, (b) composite thin films upon heating and (c) composite thin films upon cooling, Figure S4: TGA and DTG thermograms of (a-b) PCL/CaBG and (c-d) PCL/SrBG composite thin films, Figure S5. Loading-unloading indentation curves of PCL and its composite films.
