# Supplementary material for: Composite Membranes of Poly(ε-caprolactone) with Bisphosphonate-Loaded Bioactive Glasses for Potential Bone Tissue Engineering Applications
Source: Molecules. 2019 Aug 23;24(17):3067. doi: 10.3390/molecules24173067 (PMC6749304; doi:10.3390/molecules24173067)
Supplement: Supplementary file 1 [file molecules-24-03067-s001.pdf]

molecules

MDPI

Supplementary Materials

Table S1. Particle size and PDI measured by DLS.

Sample	Size (nm)	PDI
CaBG	235.7±26.11	0.547
SrBG	408.3±71.28	0.719

Table S2. Average mass and thickness values of the produced thin films.

Sample	Mass (mg)	Thickness (µm)
PCL	1.926±0.55	9±3.16
PCL/CaBG	0.758±0.35	7.5±4.86
PCL/SrBG	1.075±0.50	8±3.50
PCL/CaBG-Iba	1.223±0.17	8±3.50
PCL/SrBG-Iba	0.828±0.55	7.5±3.54

*n=25

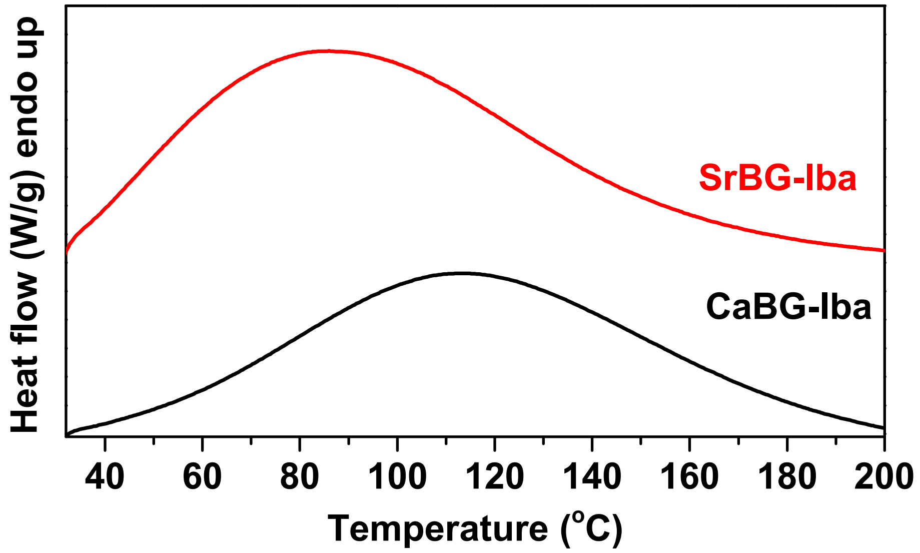

Figure S1. DSC thermograms of CaBG-Iba and SrBG-Iba during heating with 20 °C/min.

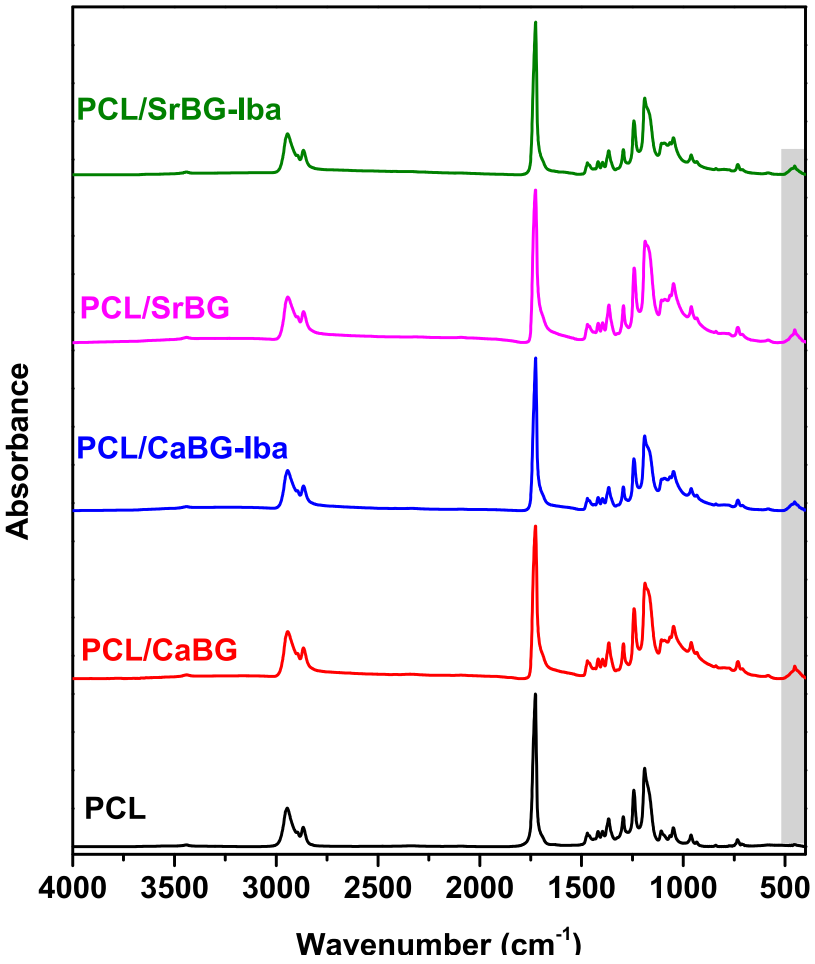

Figure S2. FT-IR spectra of the composite membranes.

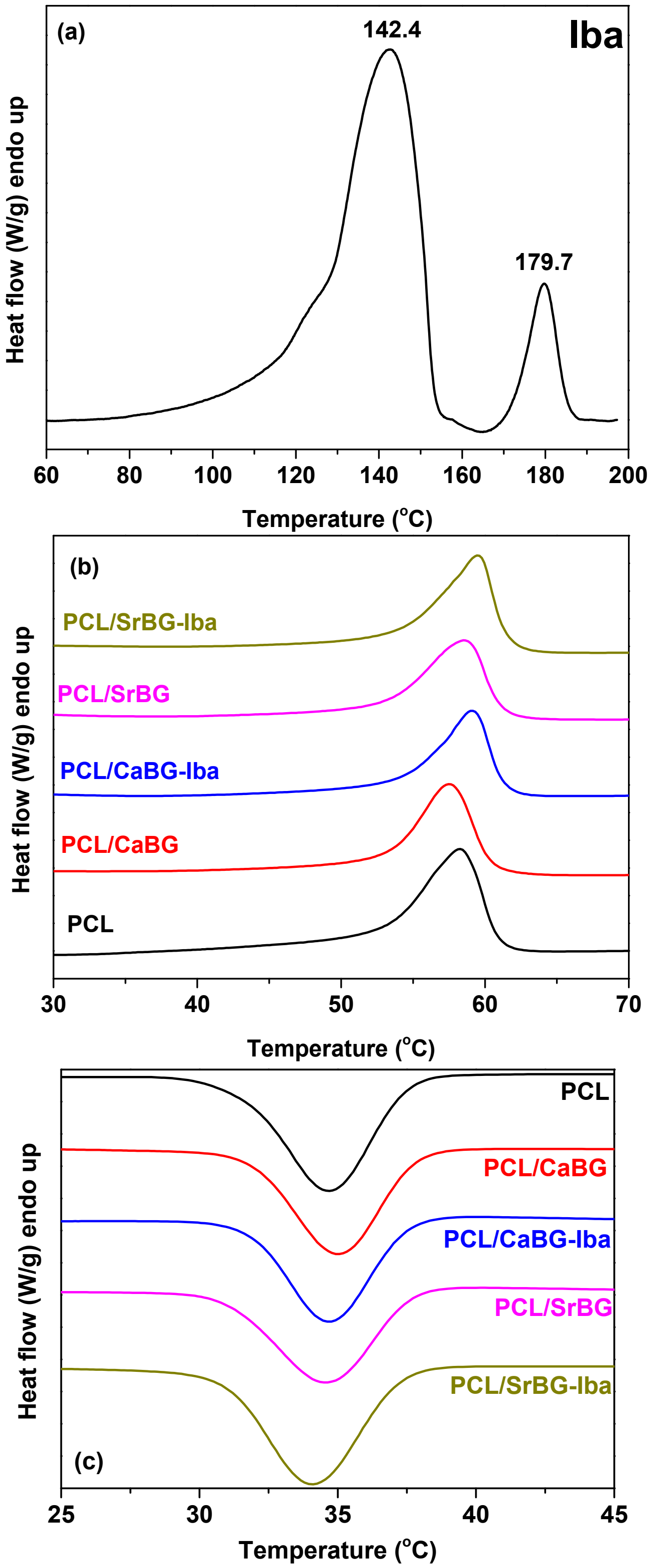

Figure S3. DSC thermograms of **(a)** Iba upon heating, **(b)** composite thin films upon heating and **(c)** composite thin films upon cooling.

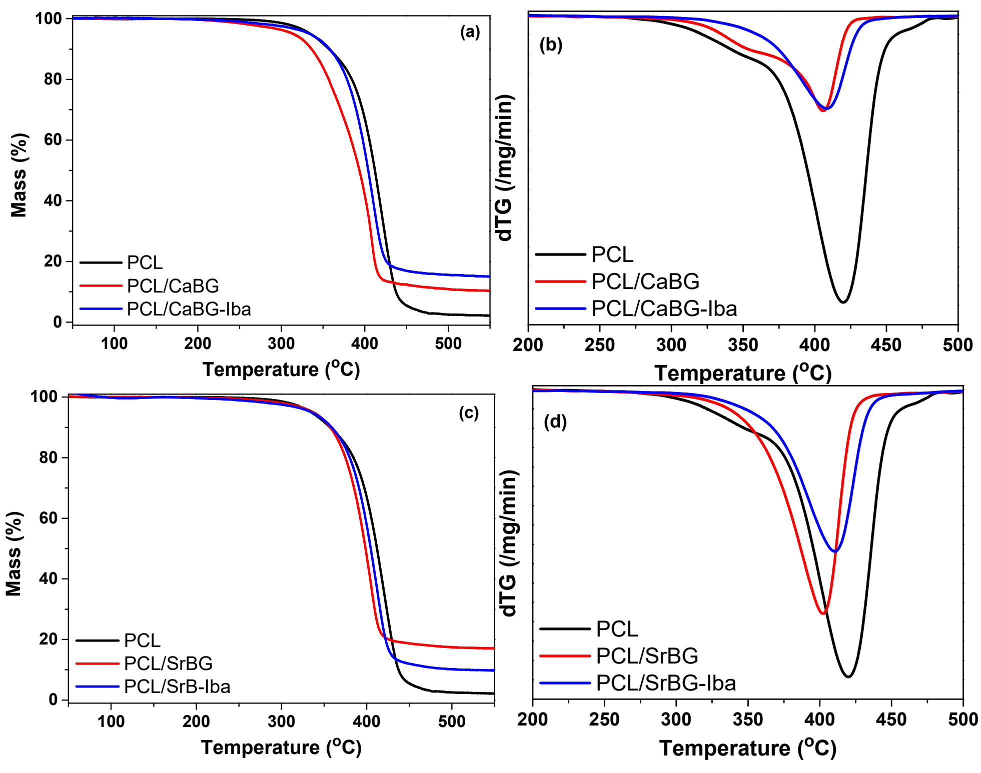

Figure S4. TGA and DTG thermograms of **(a-b)** PCL/CaBG and **(c-d)** PCL/SrBG composite thin films.

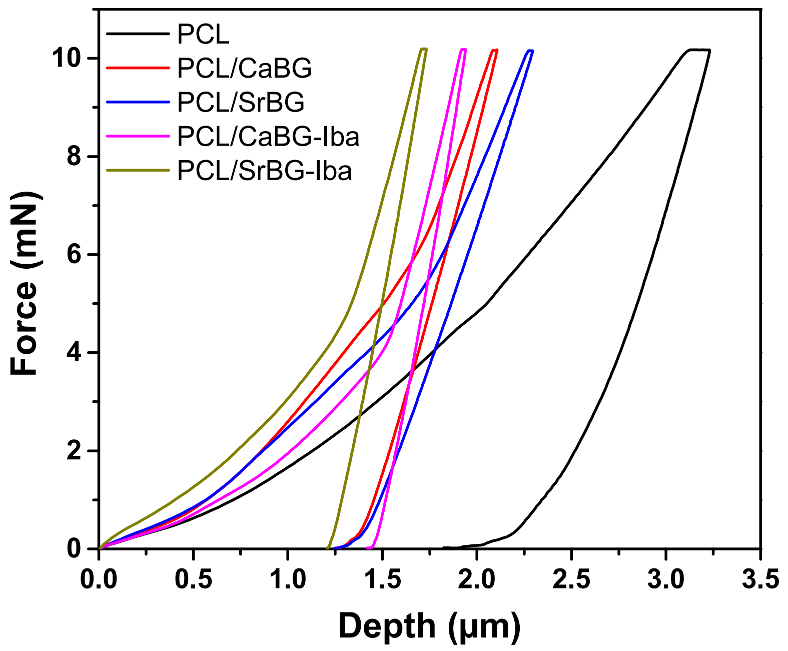

Figure S5. Loading-unloading indentation curves of PCL and its composite films.
